# A high expression ratio of RhoA/RhoB is associated with the migratory and invasive properties of basal-like Breast Tumors

**DOI:** 10.7150/ijms.43101

**Published:** 2020-10-01

**Authors:** Maud Privat, Amélie Cavard, Yanis Zekri, Flora Ponelle-Chachuat, Ioana Molnar, Nicolas Sonnier, Yves-Jean Bignon

**Affiliations:** 1 INSERM U1240 IMoST, University of Clermont Auvergne, Clermont-Ferrand, F-63000, France.; 2 Département d'Oncogénétique, Centre Jean Perrin, F-63011 Clermont-Ferrand, France.; 3 Centre d'Investigation Clinique UMR 501, F-63001 Clermont-Ferrand, France.; 4 Department of clinical research, Délégation Recherche Clinique et Innovation, Centre Jean Perrin, F-63011 Clermont-Ferrand, France.; 5 Centre de Ressources Biologiques BB-0033-00075, Centre Jean Perrin, F-63011 Clermont-Ferrand, France.

**Keywords:** RhoA, RhoB, basal-like breast cancer, BRCA1, triple negative breast cancer

## Abstract

Basal-like breast cancer is among the most aggressive cancers and there is still no effective targeted treatment. In order to identify new therapeutic targets, we performed mRNA-Seq on eight breast cancer cell lines. Among the genes overexpressed in basal-like tumors, we focused on the RhoA and RhoB genes, which encode small GTPases known to play a role in the actin cytoskeleton, allowing cells to migrate.

qRT-PCR and Western blotting were used for expression studies. Migratory and invasive properties were analysed by wound healing and Boyden chambers assays. Stress fibers formation was evaluated by fluorescent actin labeling. Rho siRNA, small inhibitor Rhosin treatment and BRCA1 transfection were performed to study the role of Rho and BRCA1 proteins.

We showed that strong expression of RhoA and low expression of RhoB was associated with the basal-like subtype of breast cancer. Decreasing RhoA expression reduced the migratory and invasive capacities of basal-like cell lines, while decreasing RhoB expression increased these capacities. Rhosin, an inhibitor of RhoA, could also reduce the migration of basal-like cell lines. Rho proteins are involved in the formation of stress fibers, a conformation of the actin cytoskeleton found in migrating cells: inhibition of RhoA expression decreased the formation of these fibers. *BRCA1*, a gene frequently inactivated in basal-like tumors, appears to play a role in the differential expression of RhoA and RhoB in these tumors, as the restoration of BRCA1 expression in a BRCA1-mutated basal-like cell line decreased expression of RhoA and increased expression of RhoB, resulting in reduced migratory capacity.

These results suggest Rho proteins as potential therapeutic targets for basal-like and BRCA1-mutated breast cancer, as migration and acquisition of mesenchymal properties are key functional pathways in these tumors with high metastatic potential.

## Introduction

Basal-like breast cancers account for 15 to 20% of breast cancers and are among the most aggressive cancers 1. These heterogeneous tumors generally do not express either estrogen or progesterone receptors, nor do they overexpress the HER2 receptor, making them “triple negative” tumors. Their gene expression profile resembles that of the myoepithelial cells of the mammary gland. Basal-like mammary tumors are very often high grade, invasive ductal carcinomas. No targeted therapy is available to date, given their lack of expression of hormone receptors and HER2 receptors 2. These basal-like cancers frequently show mutations of or loss of expression of the *BRCA1* tumor suppressor gene. The encoded BRCA1 protein maintains genomic stability through its role in the repair of DNA double-strand breaks by homologous recombination 2. Pathogenic germline mutations in *BRCA1* are responsible for approximately 20% of familial breast cancers. These tumors with *BRCA1* mutations are mainly of the basal-like subtype (69%) 2,3. However the role of BRCA1 in the development of basal-like cancers remains poorly understood 4.

Basal-like breast cancers develop metastases more frequently than other subtypes, and are thus the leading cause of breast cancer mortality 4,5. The metastatic potential of a tumor is largely due to the ability of its cells to migrate and invade the surrounding tissues. Epithelial cells acquire these abilities during an epithelial-mesenchymal transition, during which they lose their intercellular junctions and their basal-apical polarity. To migrate, the cells also rearrange their actin cytoskeleton to allow elongation and cell motility, forming lamellipodia, filopodia, invadopodia, and actin stress fibers. These cells also exert a proteolytic function allowing the degradation of the extracellular matrix. Basal-like cancer cells are thus able to invade and cross the extracellular matrix and the basal lamina to reach the circulation and spread in the body, allowing them to create metastases 6. The rearrangements of the actin cytoskeleton that occur during the epithelial-mesenchymal transition are regulated by Rho proteins, which may then be suspected of playing a role in the development of basal-like tumors.

Rho proteins (Ras homologous) are 20-25 kDa GTPases that cycle between an inactive GDP-bound form and an active GTP-bound form. This cycle is regulated by different types of proteins: GEF proteins (Guanine nucleotide exchanging factor) promote the intrinsic activity of GDP exchange to GTP; GAP proteins (GTPase activating proteins) stimulate GTPase activity, and GDI proteins (GDP-dissociation inhibitor) inhibit the dissociation of GDP from Rho proteins 7. When bound to GTP, Rho proteins have various effectors, including ROCK (Rho-activated kinases) and DRF (diaphanous-related formins) proteins, and their cooperation stimulates actin polymerization for the formation of stress fibers. These fibers are necessary to maintain the polarization and shape of the cell, and are involved in cell proliferation, motility and adhesion. As these activities are often disrupted during cancer development, Rho proteins are also involved in oncogenesis, cell survival and metastasis 7,8. Indeed, RhoA and RhoC are overexpressed in certain cancers and correlated with the aggressiveness of the tumor, i.e. the presence of metastases, and adverse clinical outcome. The role of RhoB in tumorigenesis, on the other hand, is less clear. Its expression can be increased or decreased in different cancers 9. In breast cancer, RhoB expression has been found to be inversely related to tumor progression 10. In addition, mice inactivated for RhoB appear to exhibit increased susceptibility to tumorigenesis 9.

mRNA-Seq of eight breast cancer cell lines with different phenotypes suggested that Rho proteins could be interesting therapeutic targets in triple negative breast cancer. RhoA and RhoB proteins were found to be differently expressed between basal-like and luminal cell lines 11. The aim of our study was therefore to evaluate the role of Rho proteins in the invasive properties of basal-like breast tumors. We studied the migratory and invasive capacity of different cell lines: the luminal breast cancer MCF7, and two basal-like cell lines: MDA-MB-231 with wild-type *BRCA1*, and HCC1937 with mutated *BRCA1*. RhoA and RhoB RNA silencing and Rho inhibitor (Rhosin) treatment were tested to evaluate their effect on migration and invasion. We also investigated the implication of the *BRCA1* gene in these Rho-regulated mechanisms.

## Materials and Methods

### Biological material

MDA-MB-231, MDA-MB-436, MCF7, MCF10A, T47D and HCC1937 human breast cancer cell lines were purchased from the American Type Culture Collection (Rockville, MD, USA) and grown in RPMI medium supplemented with 10% fetal calf serum, 2 mM L-glutamine and 20 μg/ml gentamicin. SUM149 and SUM1315 human breast cancer cell lines were obtained from Asterand (Hertfordshire, UK) and grown in Ham's F12 medium according to the manufacturer's instructions. SUM1315 cells were transfected with a pLXSN plasmid containing the full-length BRCA1 cDNA using Fugene 6 transfection reagent (Roche Molecular Biochemicals) to obtain SUM1315MO2. Control cells were transfected with the pLXSN empty vector. After selection in 721.5 µM G418 (Sigma Aldrich), clones were tested for BRCA1 expression by Western blotting 12. All cell lines were grown at 37 °C in a humidified atmosphere containing 5% CO2. All cell lines were stored and managed by the CJP Biological Resources Center (BB-0033-00075).

Breast tumors: archived tumor samples were retrieved for 106 women diagnosed with invasive breast cancer and treated with neoadjuvant chemotherapy protocols between 1996 and 2014 at the Jean Perrin Cancer Center. All samples were cryopreserved in the Biological Resources Center BB-0033-00075.

Three different cohorts were used to study Rho protein expression:

- Triple negative cohort 1 (n=30) was drawn from a multicentric phase II neoadjuvant study of panitumumab combined with anthracycline/taxane-based chemotherapy in operable triple-negative breast cancer (TVA) 13. Patients signed informed consent;
- Triple negative cohort 2 (n=34) was drawn from a multicentric phase II pilot neoadjuvant study of cetuximab combined with docetaxel in operable triple negative breast cancer (TENEO) (14). Patients signed informed consent;
- Luminal cohort (n=42) included residual breast tumors after treatment with neoadjuvant chemotherapy. Patients signed a form of non-opposition to research.

Study ethics approval was obtained on 17 January 2019 (CECIC Rhône-Alpes-Auvergne, Grenoble, IRB 5921).

### mRNAseq

Total cellular RNA was extracted from cell lines using RNeasy kit (Qiagen), and mRNA was purified using Oligotex mRNA kit (Qiagen). cDNA librairies were then generated following the GS-FLX Titanium cDNA Rapid Library Preparation Method Manual (Roche). Finally, emPCR amplification and 454 sequencing were performed according to the manufacturer's protocol (emPCR Amplification Manual- Lib-L LV and Sequencing Method Manual-GS FLX Titanium Series, Roche). Sequence reads were aligned on the human genome (hg19) with GS Reference Mapper software (Roche) and mapped on the human exome using an home-made software named AGSA (15). Data was then normalized by calculating the 'reads per kilo base per million mapped reads' (RPKM) for each gene. When the RPKM value was below the threshold of 0.3, then it was considered as background noise and replaced by zero. mRNA-Seq data are available in the ArrayExpress database (www.ebi.ac.uk/arrayexpress) under accession number E-MTAB-5465.

### qRT-PCR

Total RNA was extracted according to the manufacturer's protocol using RNeasy mini kit (Qiagen) for cell lines and Allprep DNA/RNA kit (Qiagen) for tumors. Quality of RNAs was checked using a 2100 BioAnalyzer (Agilent Technologies). One microgram total RNA was reverse-transcribed using First-strand cDNA synthesis kit (GE Healthcare). Multiplex quantitative RT-PCR was performed using a 7900HT Fast Real-Time PCR System (Applied Biosystems).

Predesigned and validated gene-specific probe-based TaqMan Gene Expression Assays were used, and relative gene expression was determined using the comparative threshold cycle method. Ribosomal 18S was chosen as the endogenous control gene.

### Western blot

Proteins were extracted from cells with a lysis buffer containing 50 mM Tris (pH 7.5), 5 mM EDTA, 150 mM NaCl, 0.25% Triton X-100 and 1 mM DTT. Protease inhibitors (Complete protease inhibitor cocktail, Roche) and phosphatase inhibitors (50 mM NaF, 1 mM Na3VO4) were added to the basic buffer. Twenty microgram proteins were electrophoresed on a SDS-polyacrylamide gel and transferred onto a nitrocellulose membrane. After a one-hour blocking in Tris buffered saline Tween 0.1% (TBST) containing 5% milk, membranes were incubated overnight at 4 °C with mouse monoclonal RhoA (Santa Cruz, sc-418, 1:1000), rabbit polyclonal RhoB (Santa Cruz, sc-180, 1:1000) and tubulin (Santa Cruz, 1:500) primary antibodies. Membranes were then washed three times in TBST and incubated for one hour with horseradish peroxidase-conjugated secondary antibody (1:2000). Detection was then performed with the ECL detection system (GE Healthcare).

### TCGA data analysis

Both clinical and RNA sequencing data (Illumina HiSeq RNAseq Version 2 data) of invasive breast cancers were downloaded from The Cancer Genome Atlas (TCGA) database. A total of 449 patients with information on estrogen receptor (ER), progesterone receptor (PR) and human epidermal growth factor receptor -2 (HER2) status was selected to compare gene expression profiles. 71 cases were found to have a negative ER, PR end HER2 phenotype (i.e., triple-negative), whereas 371 cases were positive for at least one of these receptors.

### RNA interference

RNA interference was performed using a pool of 4 individual siRNAs (SMARTpool ON-TARGETplus siRNA, Dharmacon). Target sequences are shown as table 1. Transfection was performed with 10nM of siRNA on adherent cells when near 60 to 80% confluence 24 h after seeding. Lipofectamine® RNAiMax (Invitrogen) was used as the transfection agent, according to the manufacturer's suggestions.

### Cell growth determination

Cells were seeded in 96-well plates, transfected with siRNA or treated with Rhosin, and cell growth was measured 48 hours later using the XTT Cell Viability Assay Kit (Biotium).

### Wound healing assay

Cells were plated and allowed to reach confluency. Twenty-four hours after transfection, a wound was made in the confluent cell monolayer by a pipette tip. The ability of cells to migrate into the cleared section was monitored. Percentage of migration was defined by three measures of lengthwise migration.

### Cell migration and invasion assays

The migration assay was performed using a Boyden chamber system (8-Am pore size, BD Biosciences). Cells were added in serum-free medium in the upper compartment of the filter. The bottom chamber was filled with complete medium. 24 h later, cells on the bottom surface of the filter were fixed, stained, and counted. This test measures the ability of cells to migrate through the pores by chemotaxis. The cell invasion assay was performed in the same conditions but with Boyden chambers precoated with Matrigel (BD Biosciences). Matrigel mimics the extracellular matrix. This test therefore evaluates cell invasion capacities to degrade this matrix and to migrate by haptotaxis.

### Immunofluorescence

Cells were plated onto glass coverslips. At 48 h after transfection, cells were fixed in methanol and permeabilized by Triton X-100 0.1%. After overnight blocking in BSA 2% - SVF 5%, cells were incubated for one hour with phalloidin-Alexa594 (Invitrogen). Coverslips were then mounted on slides with Vectashield mounting medium with DAPI.

### Statistical analysis

The mean ± SE was calculated for each data point. Differences between groups were analyzed by Student's *t* test. Data are representative of at least three independent experiments. For non-normal and heteroscedastic data (tumors' data), ANOVA was performed after elimination of outliers and log-transformation. Statistical significance was determined by one-way Welch's ANOVA.*, *P* < 0.05; **, *P* < 0.01; ***, *P* < 0.001.

## Results

### RhoA/RhoB expression ratio is higher in triple negative breast cancer cells than in luminal breast cancer cells

**Figure 1:**
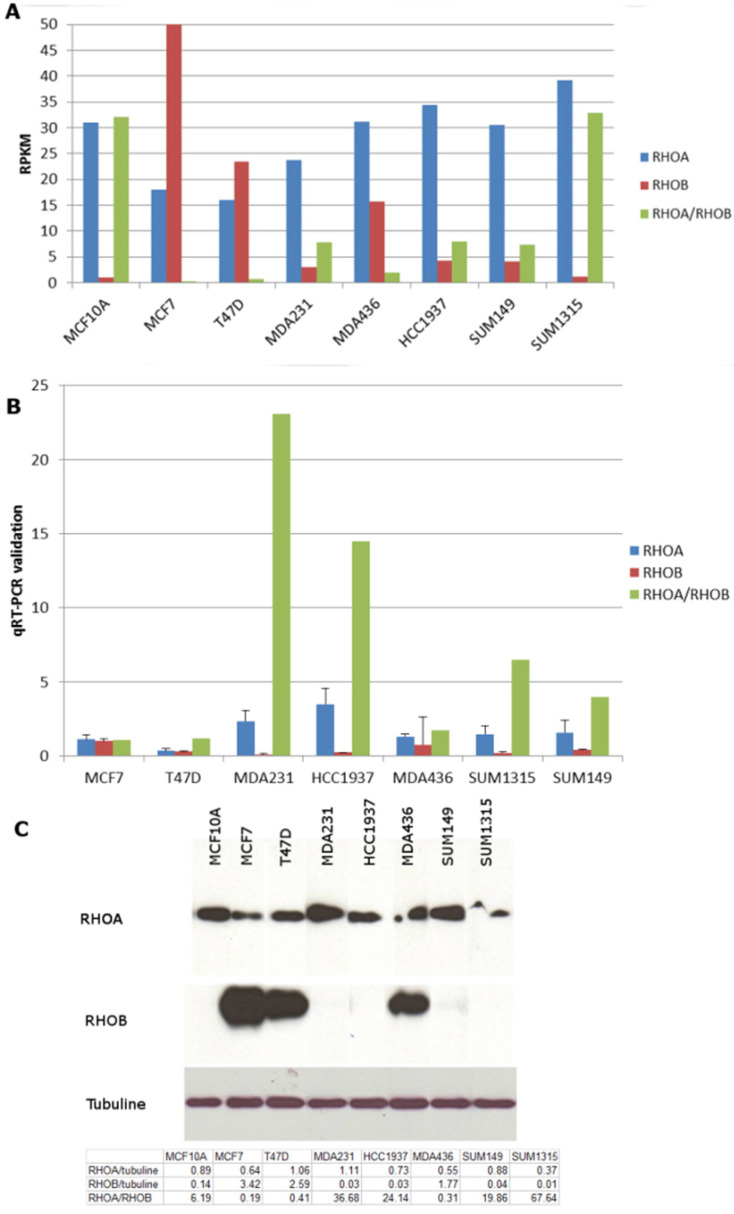
** Rho expression in triple negative breast cancer cell lines. A.** mRNA from eight breast cancer cell lines (2 luminal MCF7 and T47D, 1 non tumoral MCF10A and 5 triple negative MDA-MB-231, MDA436, HCC1937, SUM149, SUM1315) were extracted and analysed by mRNA sequencing on the sequencer GS-Flx (Roche-454). Sequence reads were aligned on the human genome (hg19) normalized in reads per kilobase per million mapped reads (RPKM). **B.** RhoA and RhoB expression in breast cancer cell lines was confirmed by q-RT-PCR. **C.** Cell proteins were extracted and Western Blot was performed using mouse monoclonal anti-RhoA (Santa Cruz) and rabbit polyclonal anti-RhoB (Santa Cruz) antibodies. Quantification of western blot was performed using ImageJ software and presented in the table under the blots.

In order to identify new therapeutic targets in triple negative breast cancer, we performed RNA-Seq of eight breast cancer cell lines, including five triple negative, two luminal and one non-tumor breast cell lines 11. The RhoA and RhoB genes were found to be expressed differently between triple negative and luminal cell lines (Figure 1A). We performed q-RT-PCR to confirm these data (Figure 1B). RhoA expression was higher in the triple-negative cell lines than in the luminal cell lines (2.7 times higher in mean, *p*=0.09). Western blotting showed that RhoB protein was strongly expressed in the luminal cell lines and was absent in all the triple negative cell lines except for MDA-MB-436 (Figure 1C). MDA-MB-436 has been classified as Basal B by Neve et al. 16 and as mesenchymal stem-like by Lehmann et al. (1): further studies will be needed to understand why this cell line behaves differently. The normal MCF10A cells behaved like the triple negative cell lines, as reported previously 16. Taken together, these data suggest a higher RhoA/RhoB expression ratio in triple negative breast cancer cells but the comparison of 2 luminal cell lines to 5 triple negative cell lines is not very statistically relevant.

**Figure 2:**
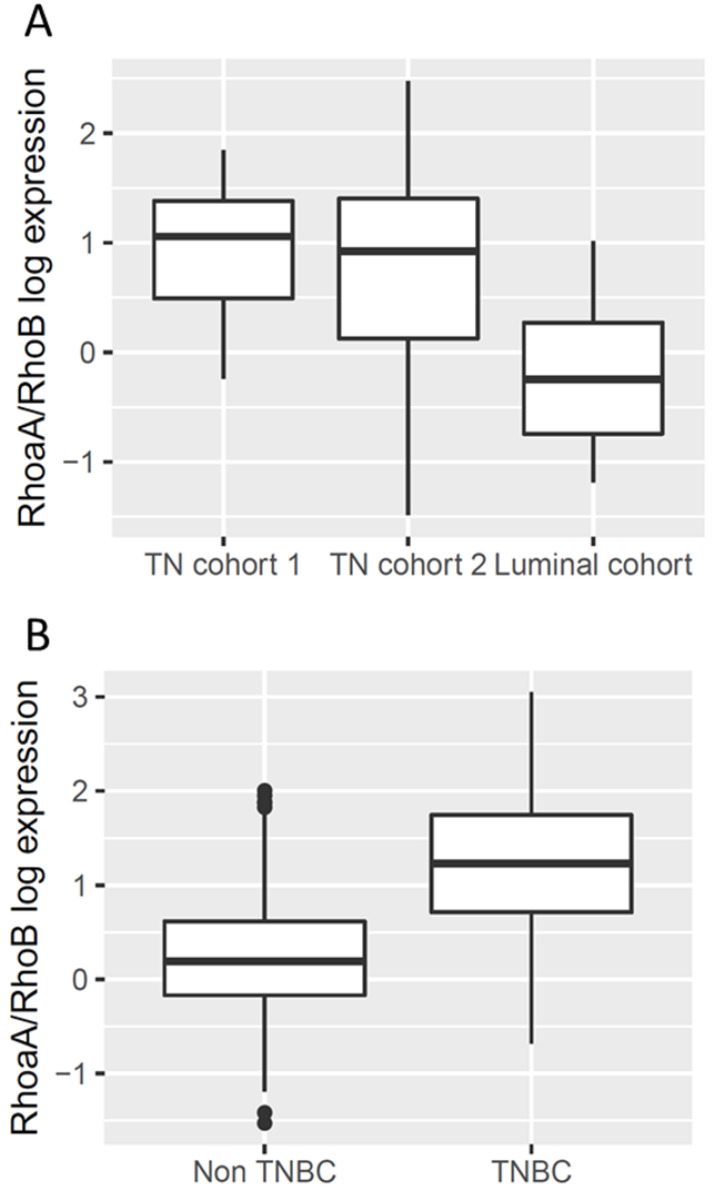
** Rho expression in triple negative breast tumors. A.** RNA was extracted from cryopreserved tumors using AllPrep DNA/RNA kit (Qiagen). Rho expression was analysed by qRT-PCR using TaqMan gene expression inventoried assay. TN: triple negative. Since data is highly non-normal and heteroscedastic, ANOVA was performed after elimination of outliers and log-transformation. There is a statistically significant difference between the three groups as determined by one-way Welch's ANOVA (Welch's F (2, 61.3) = 29.7, *p*< 0.001). Games-Howell posthoc test shows that the TN cohorts 1 and 2 differ significantly from the luminal cohort (*p*< 0.001 and *p* = 0.001 respectively); the difference between the two TN cohorts is not statistically significantly different (*p*=0.4). **B.** The Cancer Genome Atlas (TCGA) data are presented as reads per kilobase per million mapped reads (RPKM) values. TNBC: triple negative breast cancers. Again since data is highly non-normal and heteroscedastic, ANOVA was performed after elimination of outliers and log-transformation. There is a statistically significant difference between the two groups as determined by one-way Welch's ANOVA (Welch's F (1, 98) = 118.6, *p*< 0.001).

We thus tested whether this expression profile of Rho proteins (strong RhoA and low RhoB) was found in triple negative breast tumors. Archived tumor samples from 106 women from three different cohorts showed that RhoA and RhoB were both more highly expressed in luminal tumors than in triple negative tumors (Figure 2A). The RhoA/RhoB ratio was significantly higher in triple negative breast tumors (*p*<0.001) due to the large difference in RhoB expression.

Using expression data extracted from the TCGA project "Breast Invasive Carcinoma", we confirmed significant increase of the RhoA/RhoB expression ratio in triple negative breast tumors compared to other breast tumors (Figure 2B, *p*<0.001). In these data, the expression of RhoA was approximately equivalent in the two groups but RhoB was much lower in the triple negative breast cancers.

### RhoA and RhoB play positive and negative roles in migration and invasion, respectively

As RhoA and RhoB seem to have antagonistic roles in triple negative breast cancer, we studied the cellular effects of specific inhibition of RhoA or RhoB by siRNA. We used Smartpool of four different siRNA on two triple negative breast cancer cell lines (MDA-MB-231 and HCC1937) and one luminal control cell line (MCF7). Q-RT-PCR and western blotting of RhoA and RhoB showed that siRNA strongly inhibited Rho protein expression (Figure S1). RhoA inhibition induced RhoB expression, in concordance with work by Ho *et al*. 17.

Before studying the effect on cell migration and invasion, we showed that inhibition of RhoA or RhoB by RNA interference did not alter proliferation of the three cell lines (Figure S2).

**Figure 3:**
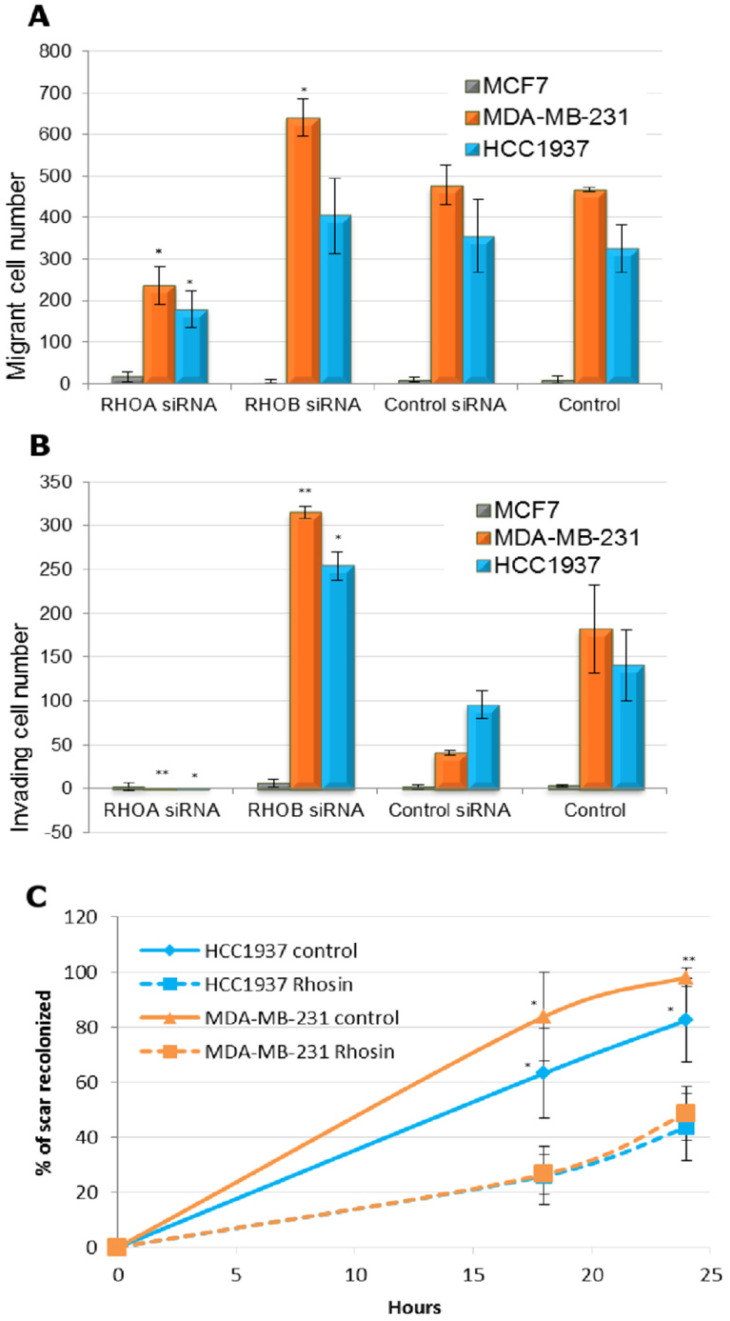
** Effect of Rho inhibition on migratory and invasive properties of triple negative breast cancer cells. A.** MCF7, MDA-MB-231 and HCC1937 cells were transfected with siRNA and migration assay was performed with a Boyden chambers system (8-Am pore size, BD Biosciences). In each cell line, Student t test was made to compare to control siRNA transfected cells. *: *p*< 0.05; **: *p*< 0.01. **B.** MCF7, MDA-MB-231 and HCC1937 cells were transfected with siRNA and cell invasion assay was performed in similar conditions, with Boyden chambers precoated with Matrigel (BD Biosciences). Forty-eight hours later, cells were fixed, stained, and counted. In each cell line, Student t test was made to compare to control siRNA transfected cells. *: *p*< 0.05. **C.** MDA-MB-231 and HCC1937 cells were treated with 30 µM of DMSO solubilized Rhosin or with 0.1% DMSO (Control). A wound was made in the confluent cell monolayer by a pipette tip. The ability of cells to migrate into the cleared section was monitored during 24 h. Percentage of migration was defined by three measures of lengthwise migration. In each cell line, Student t test was made to compare to control cells. *: *p*< 0.05; **: *p*< 0.01.

The invasive and migratory abilities of cells were tested in Boyden chambers, with or without addition of Matrigel, respectively (Figure 3A and 3B). The luminal MCF7 cells had very low migratory and invasive capacity, whereas the MDA-MB21 and HCC1937 basal-like cells showed high numbers of migrant and invasive cells. Inhibition of RhoA by RNA interference significantly decreased both the migratory and invasive abilities of the basal-like cell lines. Treatment with a RhoB-directed siRNA significantly increased invasion of both basal-like cell lines and migration of MDA-MB-231. Using an assay for wound healing (Figure S3), we confirmed antagonistic effects of RhoA (inducer) and RhoB (inhibitor) in the migratory and invasive properties of basal-like breast cancer cells, with no effect on luminal MCF7 cells.

We then tested Rhosin, a small molecule inhibitor that specifically inhibits GEF-catalyzed RhoA activation 18. This inhibitor does not affect Rac1 or Cdc42 activity but specifically targets RhoA and closely related RhoB and RhoC. As RhoB is expressed at very lower levels in basal-like breast cancer cell lines, we hypothesized that Rhosin treatment would have the same effect as RhoA siRNA. Indeed, Rhosin treatment of HCC1937 and MDA-MB-231 cells significantly decreased cell migration in wound healing tests (Figure 3C).

**Figure 4:**
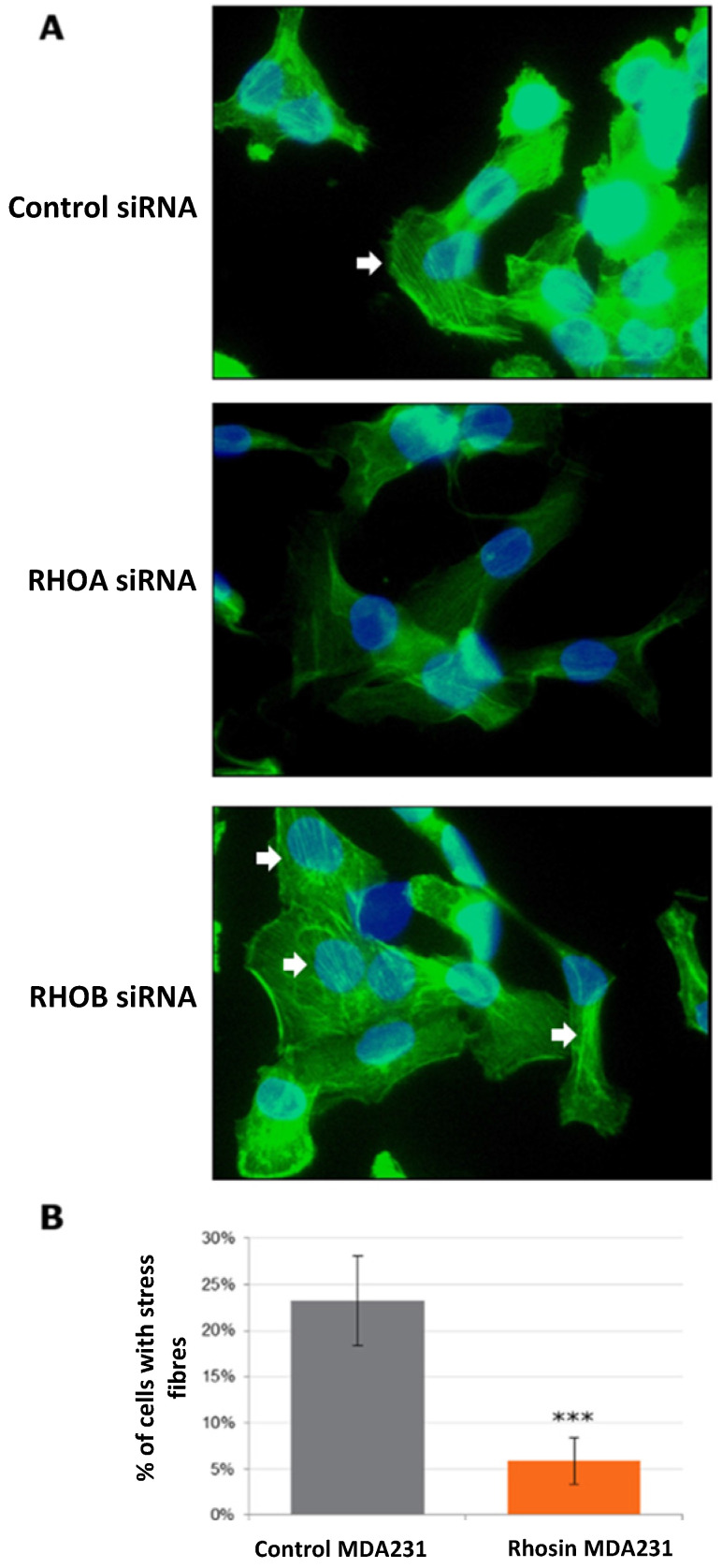
** Effect of Rho inhibition on actin cytoskeleton of MDA-MB-231 triple negative breast cancer cells.** After treatment with siRNA or Rhosin, MDA-MB-231 cells were incubated with 10 µM of Lysophosphatidic Acid for one hour to stimulate the formation of stress fibers. Cells were then fixed, permeabilized and stained with 0.5 µM phalloidin. Finally, cells were mounted with DAPI-Vectashield. A. Example of stained cells. White arrows show stress fibers. B. Photographs of several fields were taken, and the ratio of the number of cells showing stress fibers to the total cell number was performed in triplicate.

The fundamental role of Rho proteins is in the remodeling of the actin cytoskeleton, especially for the formation of stress fibers. We therefore tested whether the actin cytoskeleton was modified following treatment with Rho siRNA or with rhosin. The formation of stress fibers was activated by treatment with LPA, and cells were then stained with phalloidin, which binds to the polymerized actin filaments, preventing their depolymerization and stabilizing the cytoskeleton (Figure 4). In MCF7 and HCC1937, we did not observe stress fibers in any of the conditions tested. In MDA-MB-231 cells, we observed that RhoA inhibition by siRNA decreased the formation of stress fibers (Figure 4A). RhoB siRNA did not influence cytoskeleton organization. Rhosin treatment induced the same trend as RhoA siRNA. Quantification of the number of cells with stress fibers showed a significant 4-fold decrease after Rhosin treatment compared to MDA-MB-231 control cells (Figure 4B).

### BRCA1 gene is implicated in Rho function in triple negative breast cancer cells

**Figure 5:**
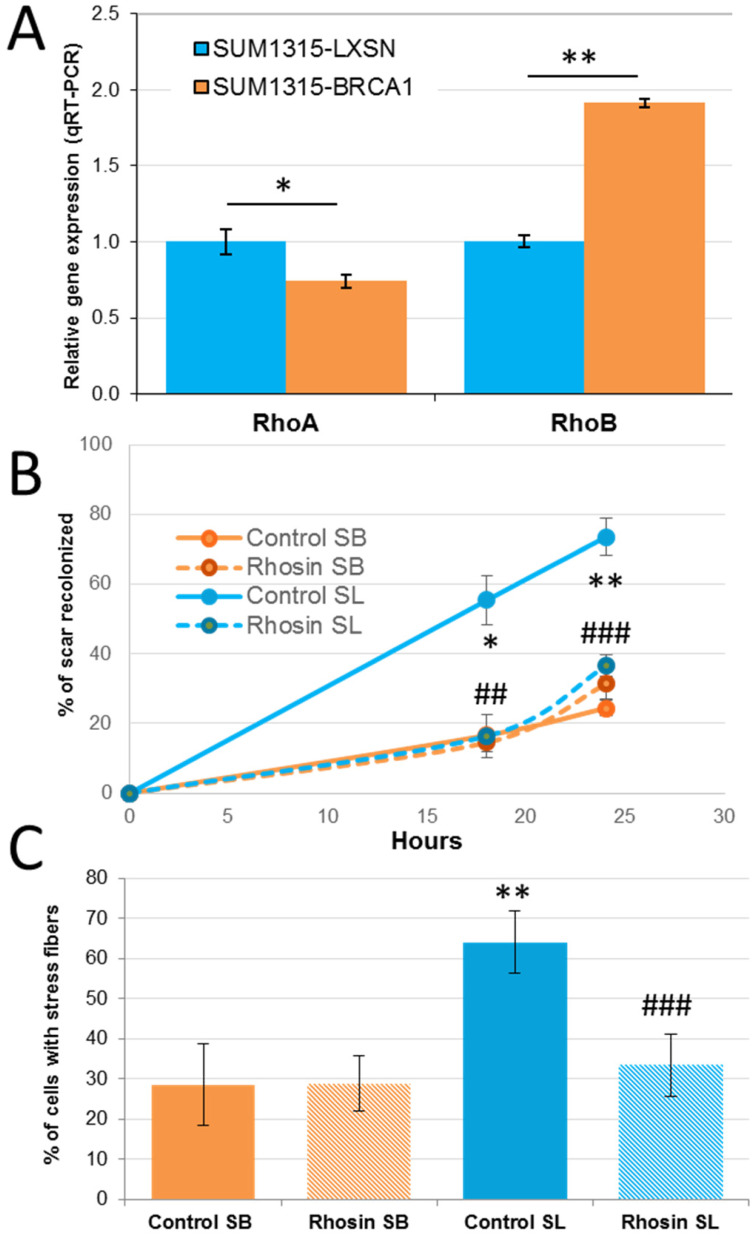
** Implication of the *BRCA1* gene in RHO function in triple negative breast cancer cells. A.** Rho expression in SUM1315-LXSN (*BRCA1* mutated) and SUM1315-BRCA1 (*BRCA1* restored) cells were analyzed by q-RT-PCR. Experiment was performed three times independently. *: *p*< 0.05; **: *p*< 0.01 **B.** SUM1315-LXSN (SL) and SUM1315-BRCA1 (SB) cells were treated with 30 µM of DMSO solubilized Rhosin (Rhosin) or with 0,1% DMSO (Control). A section of a confluent cell monolayer was produced by scratching with a p10 pipette tip. The ability of cells to migrate into the cleared section was monitored during 24 h. Percentage of migration was defined by three measures of lengthwise migration. Student's test between SL and SB cells: *: *p*< 0.05; **: *p*< 0.01; Student's test between rhosin treated and control SL cells: ##: *p*< 0.01; ###: *p*< 0.001. **C.** After treatment with Rhosin, SUM1315-LXSN (SL) and SUM1315-BRCA1 (SB) cells were incubated with 10 µM of Lysophosphatidic Acid for one hour to stimulate the formation of stress fibers. Cells were then fixed, permeabilized and stained with 0.5 μM phalloidin. Finally, cells were mounted with DAPI-Vectashield. Photographs of several fields were taken, and the mean ratio of the number of cells showing stress fibers to the total cell number was calculated. This experiment was performed three times independently. Student's test between SL and SB cells: *: *p*< 0.05; **: *p*< 0.01; student test between rhosin treated and control SL cells: ##: *p*< 0.01; ###: *p*< 0.001.

*BRCA1* gene deficiency has been associated with basal-like breast cancer. We tested whether the observed effect of Rho proteins in the migratory and invasive power of basal-like cells is dependent on BRCA1 (Figure 5). We used the SUM1315-LXSN and SUM1315-BRCA1 cell lines, which were derived from the basal-like cell line SUM1315 carrying the c.185delAG mutation of the *BRCA1* gene, stably transfected with an empty LXSN plasmid (SUM1315-LXSN) or with an LXSN plasmid coding for wild-type BRCA1 (SUM1315-BRCA1). The analysis of the expression of RhoA and RhoB by RT-qPCR in SUM1315-LXSN and SUM1315-BRCA1showed that restoration of the expression of BRCA1 inhibited expression of RhoA by almost 30% and induced expression of RhoB nearly two-fold (Figure 5A *p* = 0.016 and *p* = 5.7×10^-5^ respectively).

The migratory capacities of these two cell lines were then studied in a wound healing test. SUM1315-LXSN cells migrated much faster and much further into the scar: 24h after wounding, the SUM1315-BRCA1 cells had recolonized 21% of the scar, versus nearly 83% for SUM1315-LXSN (p = 0.007) (Figure 5B). The restoration of BRCA1 expression in the basal SUM1315 cell line seemed to be sufficient to reduce the migration capacity of this tumor cell line.

We tested whether these different migratory abilities were due to differential expression of Rho proteins. Unfortunately siRNA transfection has been shown to be highly toxic in these cell lines; we thus used the Rho inhibitor Rhosin. Rhosin had little or no effect on the SUM1315-BRCA1 cell line. This can be explained by the fact that when the BRCA1 gene is functional, RhoA levels tend to be lower, and further inhibition of RhoA does not have a large additional effect on cell migration. On the other hand, Rhosin significantly reduced migration of the SUM1315-LXSN line, since at t = 24 h, the control SUM1315-LXSN cells closed 73% of the wound, compared to 36% for the treated cells (*p* = 0.0004).

Finally we looked at stress fiber formation in control and Rhosin-treated SUM1315-LXSN and SUM1315-BRCA1 cells (Figure 5C). Regarding the role of the *BRCA1* gene in formation of stress fibers, less than 30% of the control cells of the SUM1315-BRCA1 cell line presented stress fibers, versus nearly 65% for control SUM1315-LXSN cells. In addition, fluorescent labeling of stress fibers was stronger in SUM1315-LXSN. When the SUM1315-BRCA1 cell line was treated with Rhosin, the percentage of cells showing stress fibers did not change significantly (*p* = 0.75). This is consistent with Rhosin having little influence on the migratory capacities of these cells since the presence of the BRCA1 gene reduces the expression of the RhoA protein. When the SUM1315-LXSN cells are treated, the percentage of cells exhibiting stress fibers decreased from about 65% to about 35% (*p* = 3.4×10^-7^). As a result, the decrease in the amount of active RhoA-GTP protein inhibited the formation of stress fibers.

## Discussion

Breast tumors of the basal-like subtype have an aggressive phenotype and a particularly poor prognosis, notably because of the lack of targeted treatment 4. We previously performed mRNAseq of several breast cancer cell lines and identified several genes as potential therapeutic targets for basal-like breast tumor cells 11. In the present study, we focused on small Rho GTPases and showed at the transcript and protein levels that the basal cell lines had strong RhoA expression and weaker RhoB expression, while conversely the luminal lines had strong RhoB expression and weaker expression of RhoA. To confirm these findings, we looked at the expression of RhoA and RhoB in basal-like breast cancer tumors. Using a cohort of 106 breast tumors from the Jean Perrin hospital and data from the TCGA project, we found that a high RhoA/RhoB expression ratio was characteristic of triple negative/basal-like breast cancers.

In the literature, overexpression of RhoA has been found in different tumors compared to normal tissue, often correlated with tumor aggressiveness. RhoB data are more controversial, but its expression is downregulated in several malignancies 8,19,20. In breast cancer, several studies have shown overexpression of RhoA, but also of RhoB in tumor tissue compared to healthy tissue 21. However, patients with breast cancer with overexpression of RhoB had better overall survival. Simpson et al. did not find a strong correlation between RhoA and RhoC expression and invasive potential of breast cancer cell lines 22. Nevertheless, RhoA and RhoB expression pattern has not been studied in the different subtypes of breast cancer and our study is the first to show a specific Rho expression pattern in basal-like/triple negative breast cancer.

The main function of Rho proteins is in the organization of the actin cytoskeleton, influencing cell migration 10,19,21. Basal-like tumors are usually high-grade, invasive tumors with a high rate of metastases 4, making it important to know whether Rho proteins play a role in the migratory and invasive properties of basal-like breast cancer cells. Three cell lines were chosen for our study: the MCF7 cell line from a luminal tumor, and the HCC1937 and MDA-MB-231 cell lines from basal-like tumors bearing or not (respectively) a mutation of the *BRCA1* gene. We confirmed the regulation that RhoA exerts on RhoB described by Ho *et al.* 17: inhibition of RhoA induces RhoB expression. Wound healing and migration and invasion tests showed that MCF7 cells, even when the high protein expression of RhoB was inhibited, did not migrate. It was expected that this luminal cell line has little migratory capacity. Decreasing RhoB expression was however not sufficient to induce cell migration.

In contrast, the basal-like cell lines MDA-MB-231 and HCC1937 showed both migratory and invasive capacities, which decreased after treatment with a RhoA-directed siRNA, and increased when treated with RhoB-directed siRNA. The strong migratory and invasive capacities of the basal-like cell lines could thus be linked to the strong expression of RhoA and the low expression of RhoB. This is consistent with the literature for other cancers 23-25. Huang *et al.* showed that low expression of RhoA was associated with a higher survival rate in patients with stomach cancer, and that those with strong RhoA expression developed metastases at a greater distance from the primary tumor than those with low expression. Concordant with the results for RhoB, Khalil and El-Sibai observed low expression of RhoA and RhoB in astrocytic tumors. Mokady and Meiri also showed that RhoA is overexpressed in many solid tumors, whereas RhoB acted more as a tumor suppressor with pro-apoptotic effects. A study of Rho proteins in basal-like breast cancer showed a luminal cell line derivative acquired basal characteristics while simultaneously increasing expression of RhoA protein: inhibition of RhoA by RNA interference decreased cellular migration and invasion 6. To confirm these results, it would be interesting to look at the effect of an increase in RhoB in basal-like lines, thanks to the transfection of a plasmid encoding the RhoB protein. Indeed, since the siRNA directed against RhoA induces the extinction of RhoA, but also an increase of RhoB, this experiment would make it possible to know if it is the strong expression of RhoA or the weak expression of RhoB which is responsible for important properties of migration and invasion of basal-like lines.

The effects of RhoA and RhoB inhibition on the actin cytoskeleton were also observed by phalloidin labeling. After migration stimulation, we observed stress fiber formation, especially in MDA-MB-231. Stress fibers are notably involved in the contraction of the cytoskeleton and in cell motility. RhoA siRNA induced reorganization of the cytoskeleton in MDA-MB-231 cells, leading to less stress fiber formation. These results are consistent with the decreased migratory capacities after inhibition of RhoA of these cells. However, the modulation of stress fibers could not be observed either in the HCC1937 cell line or in the MDA-MB-231 cells after inhibition of RhoB. This is probably due either to the difficulty of observing the structures of the cytoskeleton *in vitro* or to the fact that Rho proteins can act on migration and invasion independently of the cytoskeleton. This question remains to be explored.

The basal-like breast cancers more frequently have a mutation in the *BRCA1* tumor suppressor gene. To study whether BRCA1 affects migratory capacity, the *BRCA1*-mutated SUM1315 line was modified to express wild-type *BRCA1*. Restoring the expression of BRCA1 in this basal cell line was sufficient to decrease the expression of RhoA and increase the expression of RhoB, as well as to reduce migratory capacity. BRCA1 may therefore act on cell migration by modifying the expression of Rho proteins.

Rho proteins could be considered as potential therapeutic targets for basal-like breast cancers. Protein inhibition by siRNA could be very challenging *in vivo* due to siRNA instability, inefficient delivery, or incorrect bio-distribution. We thus tested a small inhibitor of Rho protein activity, Rhosin 18. This molecule contains two aromatic chemical fragments tethered by a flexible linker that binds the GEF recognition site of Rho proteins. Rhosin can thus inhibit GEF binding to Rho, preventing their activation. Rhosin is not specific to RhoA and can also bind to RhoB. Nevertheless, we showed that basal-like breast cancer cells express RhoB protein at lower levels. We thus hypothesized that Rhosin could be an interesting treatment to prevent migration and invasion of basal-like and *BRCA1*-mutated tumors. Rhosin treatment produced the same effects as RhoA siRNA in our basal-like breast cancer cell models. *In vivo* inhibition of RHO proteins by Rhosin could now be performed to confirm the therapeutic potential of this approach. A new small inhibitor of Rho proteins, Y16, could also be tested in combination with Rhosin to study the potential of Rho as therapeutic targets for basal-like and *BRCA1-*mutated breast cancer 26.

This study suggests that the targeting of Rho proteins could be an effective therapeutic strategy for basal-like and BRCA1-mutated breast cancer. This could prevent the migration and acquisition of mesenchymal properties, which are key functional pathways in these tumors with high metastatic potential.

## Supplementary Material

Supplementary figures and tables.

## Figures and Tables

**Table 1:** List of target sequences

ON-TARGETplus Human RhoA siRNA (Ref L-003860-00-0005)	ON-TARGETplus Human RhoB siRNA (Ref L-008395-00-0005)
CGACAGCCCUGAUAGUUUA GACCAAAGAUGGAGUGAGA GCAGAGAUAUGGCAAACAG GGAAUGAUGAGCACACAAG	GCAUCCAAGCCUACGACUA CAGAACGGCUGCAUCAACU CGACGAGCAUGUCCGCACA AAGCACUUCUGUCCCAAUG
