# Supplementary material for: A high expression ratio of RhoA/RhoB is associated with the migratory and invasive properties of basal-like Breast Tumors
Source: Int J Med Sci. 2020 Oct 1;17(17):2799–808. doi: 10.7150/ijms.43101 (PMC7645338; doi:10.7150/ijms.43101)
Supplement: Supplementary file 1 — Supplementary figures and tables. [file ijmsv17p2799s1.pdf]

Supporting information

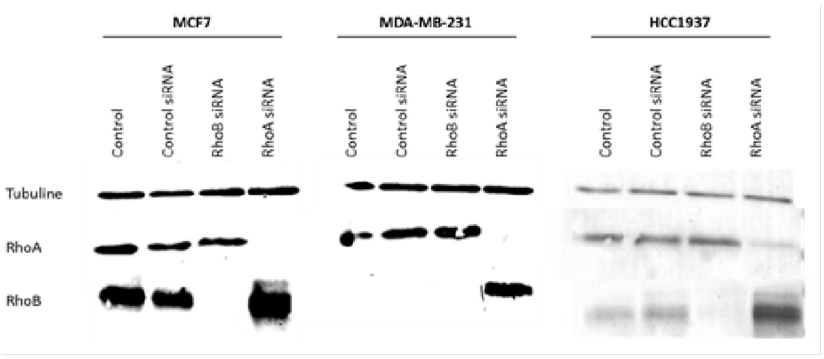

Fig. S1 Rho siRNA efficiencies

siRNA transfection of MCF7, MDA231 and HCC1937 cells were performed using Lipofectamine® RNAiMax (Invitrogen) and SMARTpool ON-TARGETplus siRNA (Dharmacon). Western blot of Rho proteins were performed to check siRNA efficiencies.

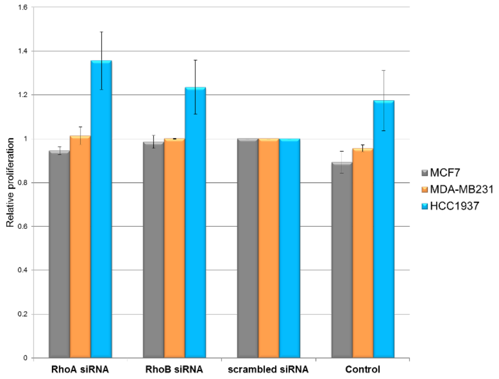

Fig. S2 Cell proliferation of MCF7, MDA-MB-231 and HCC1937 cell lines is not affected by Rho siRNAs

Cell proliferation was measured by an XTT test 72h after transfection. The values represent the average of the relative quantifications obtained from three independent experiments and the error bars represent the standard error. RhoA siRNA: cells treated with siRNA directed against RhoA; RhoB siRNA: cells treated with siRNA directed against RhoB; scrambled siRNA: cells treated with the control siRNA; Control: untreated cells.

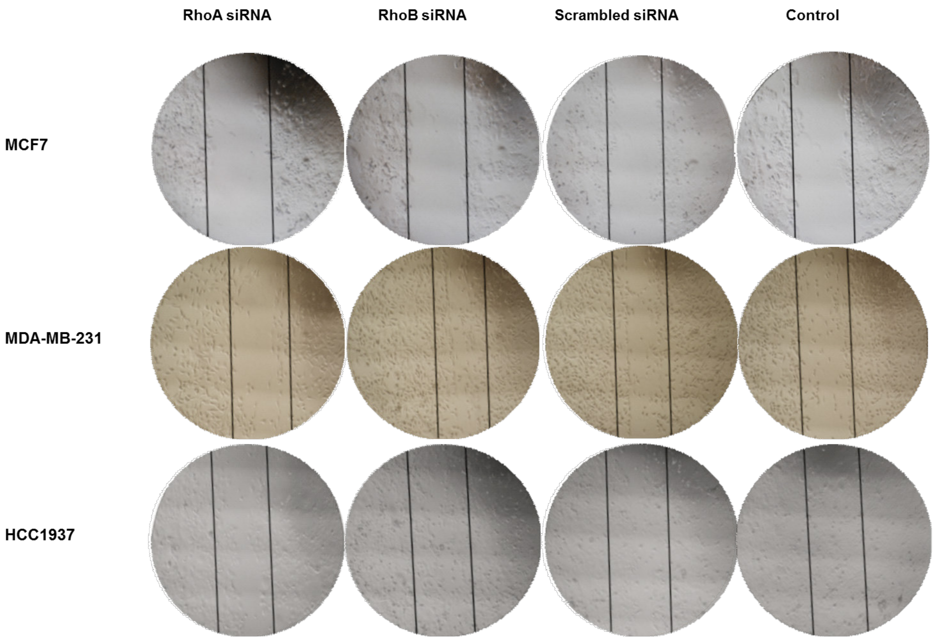

Fig. S3 Wound healing of MCF7, MDA-MB-231 and HCC1937 cell lines treated or not with Rho siRNA

An injury, represented by the black lines, was made in the lawn of the cell lines treated or not by the different siRNAs. Cell migration into the wound region was observed 20h after for MCF7 and MDA-MB-231 and 18h after for HCC1937. RhoA siRNA: cells treated with siRNA directed against RhoA; RhoB siRNA: cells treated with siRNA directed against RhoB; scrambled siRNA: cells treated with the control siRNA; Control: untreated cells. The black lines represent the initial wound made in the cells.
